# Voice analysis after cancer treatment with organ preservation

**DOI:** 10.1186/1758-3284-3-19

**Published:** 2011-04-19

**Authors:** Renata JDS Campos, Cristina TV Maciel, Marcelle G Cesca, Isabel CG Leite

**Affiliations:** 1 Instituto Oncológico of Juiz de Fora; Voice specialist; Brazilian Health Master Program of the Federal University of Juiz de Fora, Minas Gerais, Brazil; 2 Hospital Monte Sinai; Brazilian Health Master Program of the Federal University of Juiz de Fora, Minas Gerais, Brazil; 3 XXII Programa De Bolsas De Iniciação Científica Bic/Ufjf, Federal University of Juiz de Fora, Minas Gerais, Brazil; 4 Federal University of Juiz de Fora, Minas Gerais, Brazil

## Abstract

**Background:**

This cross-sectional study objects to measure, subjectively and objectively, the voice and life quality of patients with oral cavity, pharyngeal and laryngeal cancer, after organ-preservation treatment.

**Methods:**

25 cases diagnosed and treated at a high complexity oncology center in southeastern Brazil. All had oral cavity, pharyngeal or laryngeal cancer, with a therapeutic proposal of radiotherapy alone or simultaneous radiochemotherapy. Acoustic voice analysis and the Voice Handicap Index protocol were used to measure voice quality. The data were analyzed through the χ2, Student's t and Kruskal Wallis tests. Significance level was 5%.

**Results:**

After treatment, 40% complained of hoarseness, 56% complained of throat clearing, and no patient reported aphonia. On the voice quality auditory scale, 36% had moderate dysphonia. Acoustic voice analysis ranged from 184 to 221 Hz in females, and from 92 to 241 Hz in males. As for quality of life, most patients had mild physical, functional and emotional handicaps.

**Conclusions:**

Chemio-radiation organ preservation protocols in the patients studied may leave the organ but with reduced function which brings communication sequelae. In such cases, voice assessment and quality of life protocols, as well as speech therapy rehabilitation, are important tools to preserve function, measure and treat alterations, and reintegrate patients into the community.

## Background

Because several sequelae of oral communication may occur after treatment of head and neck cancer, research in this area is important to better describe the symptoms and adequately control the treated patients [1]. Costa Bandeira et al. [2], studying the quality of life and communication, voice and swallowing alterations of radiotherapy-treated tongue cancer patients, found voice and swallowing alterations and low quality of life scores after 1 year, stating the important role played by the speech therapist in the radiotherapy team.

This study aimed to analyze the voice pattern, through subjective and objective measurements, and the quality of life of head and neck cancer patients submitted to an organ-preservation protocol. The Voice Handicap Index (VHI) was used to measure voice quality [3].

## Methods

### Study population

The study was undertaken in the city of Juiz de Fora (Minas Gerais state), a reference for health care in southeastern Brazil, with an estimated population of 517,029 inhabitants [4].

It was a cross-sectional study, enrolling subjects diagnosed and treated at the Radiotherapy Division of a high complexity oncology center, during the period 2000-2006.

**Figure 1:**
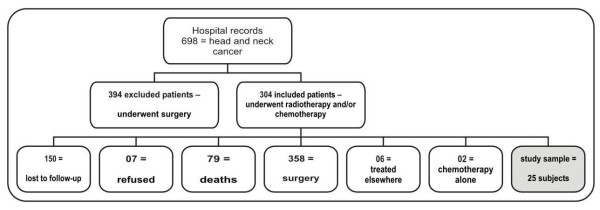
**Study flowchart**. Figure 1 shows a flowchart for patient selection. Its source is research data.

Subjects with stages T1, T2, T3 or T4 oral cavity, pharyngeal or laryngeal cancer, diagnosed and treated in the aforementioned institution, with a curative or palliative therapeutic proposal of radiotherapy alone or simultaneous radiochemotherapy, were included in the study. T4 patients were enrolled if they had declined surgery. Exclusion criteria were: death occurring previous to the study period; surgery (alone or in combination with other therapy) for head and neck cancer; story of previous neurological alteration (due to a possible interference with laryngeal physiology); physical, motor or emotional disorder precluding participation; and refusal to participate. Figure 1 shows a flowchart for patient selection. 

### Methods of study

The 25 patients included were submitted to focused interview, voice assessment (acoustic and auditory-perceptual analyses) and assessment of the VHI. All stages of voice assessment and interview were performed in a room of the local speech therapy service, by one specifically trained examiner.

Voice analysis was performed according to the parameters and method proposed by Rehder and Behlau [5]. For auditory-perceptual analysis of oral communication, we recorded a voice sample consisting of sustained emissions of the/e/vowel at standard pitch and intensity, and performed other tests of voice content. The recordings were made with a professional microphone fitted to a portable microcomputer. The subjects were assessed in the standing position, with the upper limbs extended along the body, and at a 15 cm mouth-microphone distance. 3 speech therapists, specialized in voice, and with over 3 years' experience of voice assessment, analyzed the auditory-perceptual parameters. The GIRBAS scale (G = dysphonia global grade, I = instability, R = roughness, B = breathiness, A = asthenia, S = strain) was used for auditory assessment of voice quality.

For the computerized acoustic assessment of voice sounds, the Vox Metria software was used, with the same method employed for the recording of the voice samples for the auditory-perceptual analysis. The fundamental frequency (f0) and the glottal-to-noise excitation ratio (GNE) were the measures considered for this analysis [6].

For the assessment of voice handicap, the VHI questionnaire was used, according to Moerman et al [7].

### Statistical Analysis

The data were analyzed through the χ^2 ^test with the SPSS 15.0 software [8]. Continuous variables had their means calculated, and were aligned and analyzed through Student's t test. Comparison of ordinal variables (quality of life and voice quality scales) with nominal outcomes (type of treatment, sex, tumor site) was made through Kruskal Wallis's test. Statistical significance was set at 5%.

The study met the guidelines of the Helsinki Declaration and the 196/96 Resolution of the Brazilian National Health Council [9].

## Results

Table 1 shows the sociodemographic, lifestyle and occupational features of the sample.

**Table 1:** Head and neck cancer - sample characterization, Brazil, 2009.

SAMPLE CHARACTERIZATION		
**Variable**	**Frequency**	

**Smoking**		
Yes		
Present	4	16%
Former	16	64%

No	5	20%
Daily use	15 cigarettes/day	

**Alcohol use**		
Yes		
Present	11	44%
Former	8	32%

No	6	24%
Daily intake	4 doses	

Source: Research data.

Table 2 shows tumor characterization and therapeutic approach.

**Table 2:** Tumor characterization and therapeutic approach, Brazil, 2009.

TUMOR CHARACTERIZATION		
**Variable**	**Frequency**	

**Histopathology**		
Squamous cell carcinoma	19	76%
Other types	6	24%

**UICC Stage**		
I	6	24
II	8	32
III	6	24
IV	5	20

**Tumor site**		
Mouth	3	12%
Pharynx	7	28%
Oropharynx	3	12%
Nasopharynx	1	4%
Hypopharynx	3	12%

**Larynx**	15	
Glottis	12	48%
Supraglottic	3	12%

**Therapy**		
Radiotherapy	11	44%
Radiochemotherapy	14	56%

**Treatment**		
Curative	22	88%
Palliative	3	12%

**Neck emptying**		
Yes		
Unilateral	6	24%
No	19	76%

UIIC - Universal Integrated Circuit Card.

Source: Research data.

The acoustic and auditory parameters of voice quality are shown in table 3.

**Table 3:** Acoustic and auditory analysis parameters, Brazil, 2009.

ACOUSTIC AND AUDITORY ANALYSIS PARAMETERS		
**Variable**	**Frequency**	

**Auditory analysis of voice quality**		

Hoarse	19	76%
Hoarse-breathing	3	12%
Rough	5	20%
Crepitating	4	16%

GIRBAS		
Normal	6	24%
Mild	10	40%
Moderate	9	36%

**Acoustic analysis of voice quality**		

Fundamental frequency		
Females	184	221 Hz
Males	92	241 Hz

GNE		
<50	2	8%
51 - 60	1	4%
61 - 70	3	12%
71 - 80	7	28%
81 - 90	8	32%
91 - 100	4	16%

GIRBAS - (G = dysphonia global grade, I = instability, R = roughness, B = breathiness, A = asthenia, S = strain); GNE - Glottal-to-noise excitation ratio.

Source: Research data.

None of our subjects reported professional voice use (singing or speech). Yet 36% reported voice use in the working environment and 84% in daily life conversation.

During the study period, the following frequencies referred to the year of diagnosis/treatment: 8% (2000); 20% (2002); 12% (2003); 20% (2004); 12% (2005); and 28% (2006). Our sample had no case referring to the year 2001.

79% of the subjects complained of hoarseness preceding their diagnosis. After radiotherapy, the patients complained of: throat clearing (56%), hoarseness (40%), hearing impairment (40%), and fatigue and vocal effort (8%). No patient reported aphony. As for vocal symptoms grading, 42.9% had grade I dysphonia and 47.6% had grade II dysphonia. The most frequent resonance type was laryngeal (56%). In spite of the large number of communication complaints, only 32% were referred to speech therapy.

Table 3 shows the vocal characterization of the 25 subjects who underwent organ preservation protocols.

On bivariate analysis, there was no association between acoustic analysis and sex (p = 0.385), or between acoustic analysis and GIRBAS scale (p = 0.391). GIRBAS worsened according to anatomical location (p = 0.002) and year of treatment (p = 0.017), and speech therapy increased the likelihood of GIRBAS = 1 (p = 0.048). It is noteworthy that implementation of speech therapy was influenced by sex (p = 0.057).

The main results of VHI indicated that the quality of life showed mild physical, functional and emotional handicaps. As for difficulty to be understood, 80% reported moderate difficulty, and 12% always or almost always had to repeat what they had just said, in order to be understood. Notwithstanding, 64% considered themselves talkative or extremely talkative. 40% considered their voices to have a rough pitch, 24% found their voices to be unpredictably clear, and 12% always or almost always had to make an effort to speak. In 20% the voice disappeared halfway through conversation, 8% felt embarrassed when speaking, and 8% always or almost always felt incompetent when speaking.

## Discussion

According to epidemiological data, age is one of the main risk factors for cancer development, a finding that points to an increase in cancer incidence with gains in life expectancy. 76% of our patients were males, with mean age of 64 years. Besides genetic factors, smoking and alcohol use increase the chances of head and neck cancer, with squamous cell carcinoma as the predominant histological type. In our sample, 64% were former smokers and 32% former alcohol users, with 44% present alcohol users. Mean alcohol and tobacco use time was 40 years, with 4 doses and 15 cigarettes a day, on average.

Recent studies have shown good results with organ preservation approaches that use radiochemotherapy. Although survival time is not affected, laryngeal function is not always preserved, as state Hirsch et al [10]. In our study, radiochemotherapy was used in 56% of the subjects, followed by radiotherapy alone in 44%. A 7000 Gy dose was applied to 64%, with a 5000 Gy fossa dose in 81%. Treatment was curative in 88%.

We chose to use a combination of objective and subjective measurements, according to Leeper et al [11]. Those authors concluded that, although subjective measurements of voice quality are important, objective measurements are necessary to assess subtle voice changes with time.

According to Behrman, Abramson and Myssiorek [12], 80% of prospectively studied patients had changes in their voice quality 1 year after radiotherapy. 40% of our patients still complained of hoarseness, and 56% of throat clearing, even 3 years after treatment.

Carrara-de Angelis et al. [13] concluded that of 15 patients who had received radiochemotherapy for laryngeal cancer, 33% had adequate voice quality or mild dysphonia, 40% moderate dysphonia, and 27% severe dysphonia. In a study of patients treated for glottic tumor, Caminero et al. [14] reported that 11% had normal voices, 44% had mild dysphonia, 28% had moderate dysphonia, and 17% had severe dysphonia. Those authors also reported VHI results that were close to normal. Our results were slightly better, with the following characteristic voices: hoarse (76%), hoarse-breathing (12%), rough (20%), and crepitating (16%). On the GIRBAS scale, 24% had normal voice, 40% mild dysphonia, and 36% moderate dysphonia.

Our study showed that although most subjects had mild physical, functional and emotional handicaps, several aspects of communication were impaired.

The communication problems identified in our study were: moderate difficulty to be understood, need to repeat what had been said, poor voice quality, need to make an effort to speak, and difficulty to socialize (embarrassment to speak and feeling of incompetence when speaking).

Voice quality was associated with speech rehabilitation in our study. The same finding was reported by Van Gogh et al. [15], who concluded that speech therapy was effective for patients complaining of voice impairment after treatment for early glottic carcinoma. Improvement was significant with VHI analysis, and was also confirmed through the objective voice parameters assessed.

## Conclusions

Our study results cannot be generalized, because of the small number of patients meeting the inclusion criteria and a possible survival bias that may have overestimated the perception of voice and quality of life. Yet our results suggest an important reflection on radiotherapy and its sequelae. Communication and quality of life after treatment for head and neck cancer are an important issue, once longer survival should assume satisfactory community interaction.

Our findings revealed that even after years of cancer treatment completion, there remain voice symptoms able to interfere with the quality of life. Chemio-radiation organ preservation protocols in the patients studied may leave the organ but with reduced function. Therefore, the integration of the speech therapist in the head and neck cancer treatment teams should bring techniques that will ease patient communication.

## Competing interests

The authors declare that they have no competing interests.

## Authors' contributions

RJDSC and ICGL have made contributions to conception and design of the study, acquisition of data and helped to draft and revision the manuscript. CTVM and MGC have made contributions to acquisition of data and helped to draft and revision the manuscript. All authors read and approved the final manuscript.
